# Parturition Cases1Presented at the Fourteenth Annnal Meeting of the Iowa State Veterinary Medical Association, Des Moines, Iowa, February 11 and 12, 1902.

**Published:** 1902-04

**Authors:** William Drinkwater

**Affiliations:** Monticello, Iowa


### PARTURITION CASES.[1]

By William Drinkwater, V.S.,
MONTICELLO, IOWA.

From an experience of nearly twenty years as a veterinarian in dairy districts, the writer would give some of his ideas with regard to parturition cases which test the ingenuity of practitioners of veterinary surgery.

The writer would advise the keeping of the instruments and appliances used in these cases in a convenient place, and when one of these urgent calls is to be attended to no time will be lost looking for the outfit. The instruments preferred are a repeller,

[1] Presented at the Fourteenth Annual Meeting of the Iowa State Veterinary Medical Association, Des Moines, Iowa, February 11 and 12, 1902.

or crotch; two hooks six inches long, one sharp and the other blunt pointed, each one with the eye for the rope made large enough to place a loop of rope through, thus avoiding the necessity of putting one cord in and pulling it through; two curved embryotomy knives, to cut by pulling toward the operator; a saw to sever leg bones when they cannot be turned without risk of lacerating the uterus; two small ropes, each six feet long, sash cord preferred; an injection pump; a bottle of creolin, and a large piece of tar soap. The writer has, for cases where it is difficult to keep the patient in a standing position or where the animal is lying down and needs to be raised, a set of pulleys, which consists of one double pulley, to attach to a pole or beam overhead in the stable, and two single pulleys; also forty feet of rope, one-half inch in diameter, rigged by passing twice through the double pulley and once to each single pulley, which is attached to loops of rope passed inside of each thigh of the mare or cow. The last, and a very important part, is a pair of waterproof overalls, a pair of rubber overshoes, and an old coat with the sleeves cut off at the shoulders, with an elastic cord run around the armholes to draw them in, so as to protect the clothing of the operator.

We are called in only after the owner or someone else has failed to relieve the distressed animal, and we must be prepared for hard work. It is usually well to dress for the operation first of all, as when one gets started he may find what appears to be simple very difficult before he is done with it. First, lather the hands and arms well with the tar soap, and wash out the vagina and as much of the uterus and fœtus as possible with a creolin douche by aid of the injection pump, and examine carefully all around the fœtus and walls of the uterus for any lacerations which may have been made by someone who has failed to relieve the dystocia. If a rupture or large laceration is found, inform the owner at once and advise him that further work is useless, as it is a rare case that survives such lesions; but if he insists that you remove the fœtus this may be done, but no hope of the recovery of the mother should be given.

What the difficulty with the fœtus may be is for the operator to determine. He must use his best judgment in rectifying the position. He should be deliberate and careful in his action, avoiding too great haste. He should make every move count, and use great care not to bruise, lacerate, or rupture any of the parts of the mother. When ready to extract, the cords may be applied to the legs and considerable force used. If the fœtus is dead the hooks may be inserted where the firmest holds are found. When the fœtus has been dead for a considerable time it is generally bloated and too large for extraction; then the hooked knife may be passed up under each of the presenting legs, the skin ripped toward the operator, the hand pushed up under the skin, separating it from the muscles, the rope attached, and the limb pulled off the body. Sometimes the abdomen may be ripped open and the intestines removed, to reduce the size of the fœtus. If the reduction is made and most of the skin is left it may be pulled into the vagina and ropes or hooks attached to the skin or solid parts, and the bystanders may be given something to do. When removal is accomplished, a pail of warm, soft water, with creolin enough added to make it quite white, should be at hand, and the uterus flooded. If the patient does not expel it in a few minutes the hose can be taken off the injection pump, one end placed in the uterus, and the other end dropped on the stable floor or gutter, and the fluid thus siphoned out. This may be repeated. If the placenta has not loosened enough to be removed you may direct the owner to inform you if it is not expelled by the next day, and you can return and remove and wash out the uterus again. Last of all, to smear the vagina and sore parts with pure creolin.

The writer would not attempt to describe all the malpresentations and deformities that are met with in veterinary obstetrics, and does not assume that he is an expert in parturition cases, but gives some of his ideas of the managing of these cases, and hopes to bring up some points for discussion which may be of benefit to the members of this Association.

### Discussion.

Dr. Heck said that the removal of a putrid fœtus is a very difficult operation. He questioned if the undertaking would not be facilitated by injecting into the fœtus a quantity of a strong disinfecting and deodorizing substance, as, for instance, formaldehyde or carbolic acid. He said that the injection of a carbolic acid solution into the uterus of a sow containing putrid fœtuses will overcome the bad odor and render the fœtuses much easier of removal.

Dr. B. H. Miller and Dr. Scott thought that creolin was not to be recommended for use as a uterine douche, as it is apt to bring on straining.

Dr. Lyford recommends the use of some bland oil, as linseed oil or cottonseed oil, in large quantities, to be pumped into the uterus and vagina in cases where the fœtus is dry and hard to extract. One to three gallons may be needed. In one case he used ten gallons. Raw eggs in large quantities make a good lubricant, and may be used if available and expense does not forbid.

Dr. Scott uses lard in large quantities as a lubricant.

